# Assessment of Genetic Diversity among *Pleurotus* spp. Isolates from Jordan

**DOI:** 10.3390/jof4020052

**Published:** 2018-04-29

**Authors:** Hanan Aref Hasan, Ahmad Mohamad Almomany, Shireen Hasan, Ayed M. Al-Abdallat

**Affiliations:** 1 Department of Plant Production and Protection, Faculty of Agriculture, Jeresh University, Jeresh 26150, Jordan; jarunih@yahoo.com; 2 Department of Plant Protection, Faculty of Agriculture, The University of Jordan, Amman 11942, Jordan; momanyah@ju.edu.jo; 3 Hamdi Mango Center for Scientific Research, The University of Jordan, Amman 11942, Jordan; s.hasan@ju.edu.jo; 4 Department of Horticulture and Crop science, Faculty of Agriculture, The University of Jordan, Amman 11942, Jordan

**Keywords:** *Pleurotus*, mushroom, inter simple sequence repeat (ISSR), internal transcribed spacer region, genetic diversity

## Abstract

*Pleurotus* is considered an important genus that belongs to the family *Pleurotaceae* and includes the edible King Oyster mushroom (*Pleurotus eryngii*). In the present study, 19 *Pleurotus* isolates were collected from two locations in the north of Jordan (Tell ar-Rumman and Um-Qais). The morphological characteristics among collected isolates revealed that there was a morphological similarity among the collected isolates. Nucleotide sequence analysis of the internal transcribed spacer (ITS1–5.8S rDNA–ITS4 region) and 28S nuclear large subunit (nLSU) in the ribosomal DNA gene of the isolated stains showed that all of them share over 98% sequence similarity with *P. eryngii*. Genetic diversity among the collected strains was assessed using inter simple sequence repeat (ISSR) analysis using 18 different primer pairs. Using this approach, 141 out of 196 bands obtained were considered polymorphic and the highest percentage of polymorphism was observed using primer UBC827 (92.3%) with an overall Polymorphism Information Content (PIC) value of 70.56%. Cluster analysis showed that the Jordanian *Pleurotus* isolates fall into two main clades with a coefficient of similarity values ranging from 0.59 to 0.74 with a clear clustering based on collection sites. The results of the present study reveal that molecular techniques of ISSR and rDNA sequencing can greatly aid in classification and identification of *Pleurotus* spp. in Jordan.

## 1. Introduction

*Pleurotus eryngii* or King Oyster mushroom is a tetra-polar heterothallic edible fungus, which can form an edible fruiting body on lignocellulosic substrates [1]. It belongs to the phylum *Basidiomycota*, class *Agaricomycetes*, order *Agaricales*, family *Pleurotaceae*, genus *Pleurotus* [2]. This edible mushroom is distributed from central Europe to the northern coast of the Mediterranean Sea, North Africa, Central Asia, Turkey, and Iran [3,4,5]. *P. eryngii* is among the most cultivated species of all *Pleurotus* due to its excellent shape, consistency of cap and stem dimensions, culinary qualities, and its long shelf life [4]. Moreover, *P. eryngii* has important medicinal properties due to its immune-stimulatory effects and antifungal activity [6,7]. Moreover, it can be used in the degradation of raw plant materials, bioremediation of heavy metal contamination in soil, and bioconversion of organic compounds [8].

In nature, there are ~1150 edible and food fungal species available, from which, 40 species belong to *Pleurotus* [9]. The breeding of new *P. eryngii* varieties with superior properties, such as high productivity and disease resistance, is still urgently needed. Meanwhile, a drastic loss of genetic diversity in natural populations of *Pleurotus* is occurring as a consequence of urbanization, domestication, and loss of habitat [10]. Therefore, assessment of genetic diversity among local *P. eryngii* isolates is crucial for the improvement of *P. eryngii* and future preservation of its valuable germplasm [1]. For this purpose, molecular markers are commonly used for the analysis of genetic diversity and they revealed superiority over conventional traditional methods of classification such as morphological traits and isozymes [11]. For instance, internal transcribed spacer (ITS) regions of the ribosomal RNA genes have been widely used to reconstruct the phylogenetic history of several fungi at the upper and lower levels of relatedness [12,13,14]. For *P. eryngii*, RPB2 seems to be more accurate to distinguish varieties [15], but ITS regions are useful to compare new samples to the different known species. Molecular markers are independent of environmental parameters and provide high levels of detectable polymorphism to determine the genetic diversity that can be used as an efficient tool in breeding programs purposes [16,17]. Several molecular markers have been used in *P. eryngii* diversity studies such as Randomly Amplified Polymorphic DNA (RAPD), Inter Simple Sequence Repeat (ISSR), and Sequence Related Amplified Polymorphism (SRAP) [8,17].

The aim of this study was to identify, using morphological and molecular techniques, several *Pleurotus* spp. Jordanian isolates, collected from two different locations. Molecular identification of the Jordanian *Pleurotus* isolates was achieved using sequence information of the internal transcribed spacer (ITS1–5.8S–ITS2) and the 28S nuclear large subunit (nLSU) in the nuclear ribosomal DNA. Secondly, the genetic diversity among Jordanian *Pleurotus* isolates was assessed using inter simple sequence repeat (ISSR) markers.

## 2. Materials and Methods

### 2.1. Collection and Pure Culture Preparation

Nineteen *Pleurotus* isolates were collected in February 2014 from two different locations across Jordan: Tell-ar-Rumman (Latitude: N 32°16′40″; Longitude: E 35°83′41″; Altitude: 575 m) and Um-Qais (Latitude: N 32°65′35″; Longitude: E 35°68′54″; Altitude: 252 m). The two locations were selected as they are parts of the Mediterranean climatic zone in Jordan where forest and vegetation cover exist, which represent a natural habitat for *Pleurotus.* The isolates were collected in valleys behind streams of water very close to rivers; Tell-ar-Rumman isolates were near the Zaraqa River and Um-Qais isolates were near the Jordan River. Such areas are known to have suitable humidity, sufficient rainfall, fertile soil, and temperatures to promote *Pleurotus* growth. The isolates were collected behind Umbelliferae plants; Um-Qais isolates were found only on *Ferula armandii*, while, in Tell-Rumman, the first 7 samples were found on *Ferula armandii* and the last three were found on *Daucus carota* subsp. s*ativus*.

Pure mycelium colonies from the collected *Pleurotus* isolates were obtained by two methods: spore print and from mushroom tissue. The fruit body of the collected *Pleurotus* was cleaned from any plant and soil debris, then it was dried well on white filter paper for 24 h. The next day, spores were isolated with a sterile needle and then inoculated into Potato Dextrose Agar (PDA) sterile autoclaved media. Another method used to obtain mycelium was by cutting the collected mushroom bodies into very thin small pieces that include the cap, gills, and the stem of the strain. Then, these small cut pieces were moved gently into a clean petri dish with sterile water to clean them from any soil and plant debris, and were then transferred into another washing petri dish with 0.5% sodium hypochlorite for 3 min where they were removed gently and washed in sterile water. After that the washed pieces were dried on white filter paper and used to inoculate PDA autoclaved media to get the necessary mycelia colony of *Pleurotus* spp. After four days of incubation, newly emerging mycelia was used to establish a pure culture on PDA media, which was maintained at 25 °C in the dark. For storage, new emerging mycelia was picked and transferred to PDA media and kept at 4 °C in the refrigerator.

### 2.2. Morphological Characterization

For morphological characterization, each isolate was studied in the laboratory by taking measurements of the cap (shape, size, color), and the texture (smooth, fibrous, sticky, and scaly), stem (height, diameter, color and texture (ring, volva, basal bulb, and roots), gills (color, shape, and attachment to stem), flesh (color, texture, exude milk, smell and taste), spore (color, shape, spore print, and measurements of length and width). The procedures for morphological descriptions were followed according to [18,19], and by using the Fungal Databases Nomenclature and species Banks, and by referring to https://www.mushroomthejournal.com.

Spore print for each fresh mature sample was also defined by laying the fruiting body over glass sheath and by covering it overnight with a beaker to avoid drying. Spores of each mushroom body were microscopically (Labphoto, Nikon, Tokyo, Japan) investigated and photographed recording the shape, color, and size as described in [20]. 

### 2.3. Dna Extraction

Total Genomic DNA was extracted from young, healthy and fresh stem and cap tissue samples from collected fruiting bodies using E.Z.N.A. ^®^ SP Fungal DNA Mini Kit (Omega Bio-tek, Inc., Norcross, GA, USA) following the manufacturer’s instruction. The DNA quality was assessed by gel electrophoresis in a 1% agarose gel stained with Red Safe (Intron, Bio-tek, Seoul, Korea). DNA concentration was determined using a spectrophotometer (BIO-RAD, Smart spec^Tm^ plus spectrophotometer, Hercules, CA, USA) and stock solution (30 ng/µL) for each isolate was prepared with sterile distilled water and then stored at −20 °C for PCR reaction.

### 2.4. ITS and Nlsu Amplification and Sequence Analysis

For the amplification of the ITS region, the ITS1 forward (5′-TCCGTAGGTGAACCTGCGG-3′) and ITS4 reverse primers (5′-TCCTCCGCTTATTGATATGC-3′) were used as described previously [21]. For the amplification of the nLSU region, the LR0R (5′-ACCCGCTGAACTTAAGC-3′) and LR16 (5′-TTCCACCCAAACACTCG-3′) were used as described previously [22]. The PCR amplification was carried out in a 25 µL total volume reaction consisting of 12.5 µL PCR master mix (Intron, Bio-tek, Seoul, Korea), 1.25 µL of 10 pmol of both primers, 2.5 µL of template DNA from the stock (30 ng/µL), and 7.5 µL nuclease- free water. The PCR program conditions were as follows: 94 °C for 5 min for initial genomic DNA denaturation, 40 cycles of 94 °C for 40 s, 55 °C for 30 s, and 72 °C for 1 min, and a final elongation step at 72 °C for 5 min. The amplified products were purified using E.Z.N.A Gel Extraction Kit-Spin (Omega Bio-tek, Inc., Norcross, GA, USA), following the manufacturer’s instructions, and the products were analyzed to test the integrity and concentration on 1.5% agarose gel using electrophoresis.

PCR products were directly sequenced from both directions using the same primers used for the amplification. Final cleaned PCR products were sent to Macrogene Inc. (Seoul, Korea) and were sequenced using an ABI 3730XL capillary electrophoresis sequencing station (Applied Biosystem, Foster City, CA, USA). For molecular identification, the obtained DNA sequences from the Jordanian isolates were used as a query in a BLASTn (http://blast.ncbi.nlm.nih.gov) search against the GenBank nr DNA sequences database as described previously [23]. DNA sequences of the Jordanian isolates were deposited in the GenBank under PopSet: 1374951795 (Accession numbers: MH168609.1 MH168630.1).

Phylogenetic analysis was carried out using MEGA 6 software [24]. The ITS sequences of the 19 *Pleurotus* isolates from this study was used, together with previously published ITS *Pleurotus* spp. reference sequences, including *P. eryngii* (EU395845.1), *P. eryngii var. ferulae* (AB286152.1), *P. nebrodensis* (AB286148), *P. eryngii var. tuoliensis* (AB286164.1), *P. ostreatus* (AY450345), and *P. cystidiosus* (AY315766). The sequences were aligned using the Muscle algorithm and used to build a phylogenetic tree by calculating distance matrices for neighbor-joining analysis with the Kimura two-parameter model and bootstrapping analysis with 10,000 replicates to test the robustness of the internal branches.

### 2.5. Issr Analysis

Eighteen ISSR primers were selected and used in this study based on sequence information described in [8,17]. The ISSR amplifications were carried out in a 25 µL reaction containing 2.5 µL of 30 ng DNA template, 0.25 µL primer (10 pmol), 12.5 µL master mix (Intron, Bio-tek, Seoul, Korea), and nuclease-free water. Amplification conditions were as follows: an initial denaturation at 94 °C for 5 min, followed by 35 cycles each at 94 °C for 30 s, 41 °C for 45 s, and 72 °C for 90 s, followed by a final extension for 7 min at 72 °C [8,17]. The PCR products were visualized using 2% agarose gel using a high resolution gel electrophoresis, (Elchrom Scientific Ag, Cham, Switzerland) stained with red safe dye (Intron, Bio-tek, Seoul, Korea). The ISSR analysis was repeated three times for each sample and only reproducible bands in the three replicates were used in further analysis.

For genetic diversity assessment, each ISSR reproducible band was scored and transformed into a binary matrix where the presence of a reproducible polymorphic DNA band at a particular position (obtained from three technical replicates) on the gels was scored as 1, while 0 means its absence. The collected marker data was used to generate a distance matrix using the Numerical Taxonomy Multivariate Analysis System (NTSys-PC applied Biostatics, version 2.02) [25], a program based on the Dice similarity coefficient [26]. A dendrogram was generated based on the UPGMA algorithm (Un-weighted pair group method with arithmetic average) as described previously [8].

## 3. Results

### 3.1. Morphological Characteristics of Jordanian Pleurotus Isolates

**Figure 1:**
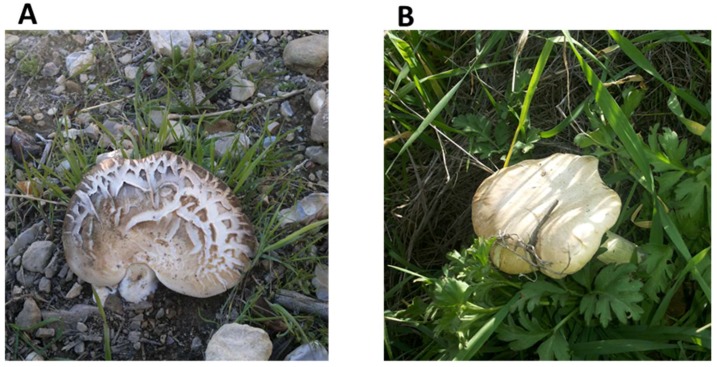
*Pleurotus* isolates collected from two different natural habitats across Jordan. (**A**) Tell ar-Rumman; (**B**) Um-Qais.

All collected *Pleurotus* isolates from the two locations were examined macroscopically and microscopically and their morphological characteristics were recorded depending on descriptors available for *P. eryngii* (Figure 1 and Table 1). The characteristics among the collected *Pleurotus* isolates from the same location revealed that there are variations at the morphological level (Table 1). There were differences between cap color and diameter among isolates collected from the two habitats and in some instances within the same location (Table 1). For instance, the stem shape was different between collected isolates from the two locations; it was thick cylindrical in Tell ar-Rumman samples, while it was thick and eccentric in the Um-Qais samples (Table 1). Also, clear differences were obtained in gills color of different isolates; it was whitish honey in the Tell ar-Rumman samples but it was greyish to white in the Um-Qais sample sites. Microscopically, the length and width of spore were different among isolates per habitat. For instance, Tell ar-Rumman samples had the largest spores’ length (range between 5 and 10 µm) and width (range between 2 and 5 µm) when compared to samples from Um-Qais (Table 1).

### 3.2. Molecular Identification Using ITS Analysis

**Figure 2:**
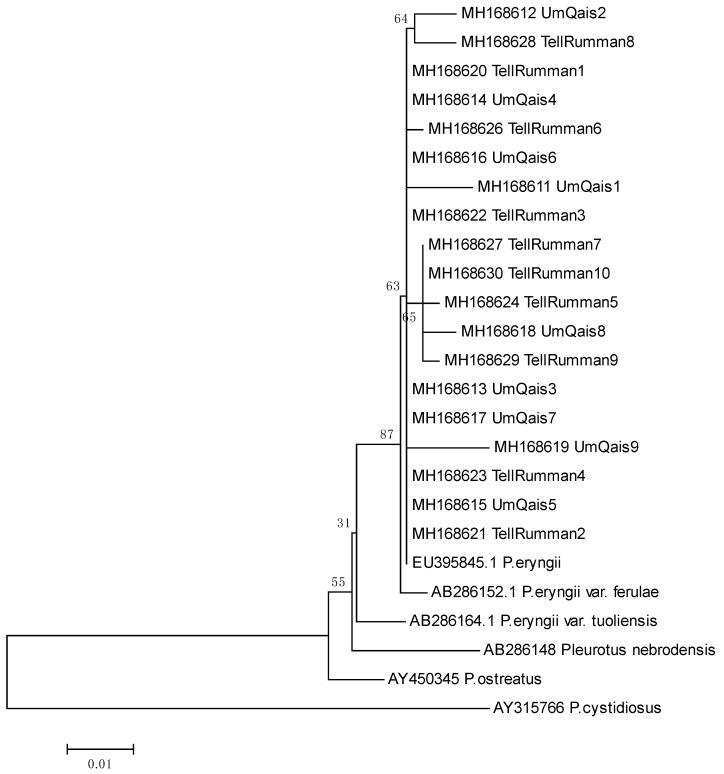
Phylogenetic tree using neighbor-joining analysis between 19 *Pleurotus* Jordanian isolates collected from two locations (Um-Qais and Tell ar-Rumman) based on ITS sequences and selected reference sequences (GenBank accession number for collected isolates and reference strains are given before the isolate name).

For ITS and nLSU sequence analysis, the amplification products from different *Pleurotus* isolates gave band size around 600 bp and in all cases a single sharp band was produced that was used for direct sequencing. The partial ITS and nLSU nucleotide sequences of the 19 *Pleurotus* isolates were obtained and analyzed for sequence similarity in the GenBank using the blastn tool (Table 2). All collected isolates produced high sequence similarity percentage (above 98%) against nLSU sequences from previously deposited sequences of *P. eryngii*. All collected isolates produced high sequence similarity percentage (above 97%) against ITS sequences from previously deposited *P. eryngii* sequences. Phylogenetic analysis with ITS sequences of the 19 isolates with high similarity to *P. eryngii* with different *Pleurotus* spp. reference sequences confirmed the identification of the collected isolates within the *P. eryngii* var. erygnii-ferulae complex [27] (Figure 2).

### 3.3. Genetic Diversity by Using ISSR

**Figure 3:**
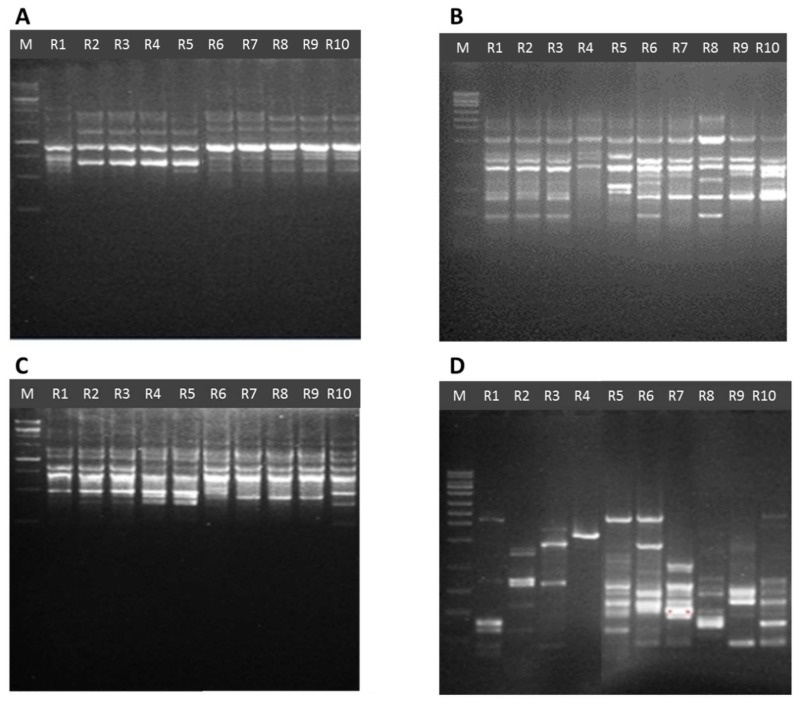
Representative inter simple sequence repeat (ISSR) profiles in 11 *Pleurotus* Jordanian isolates from the Tell ar-Rumman location. (**A**) UBC866; (**B**) UBC864; (**C**) UBC809; (**D**) UBC823; M: 1 Kb Ladder (Gene Direx, Keelung City, Taiwan).

Eighteen ISSR primers previously tested on *Pleurotus* isolates from different regions [8,28] were used to assess genetic diversity among the Jordanian isolates (Figure 3; Table 3). Seventeen ISSR primers gave reproducible results in at least three independent reactions as shown in Table 3. The UBC811 failed to produce any PCR products and was discarded from further analysis. From the total number of bands obtained and the percentages of polymorphisms of each tested primer, 141 out of 196 bands were considered polymorphic, generating a PIC overall average of 70.56%. The maximum percentage of polymorphism was observed using primer UBC827 (92.3%), while the lowest percentage was observed using primer UBC809 (6.6%).

**Figure 4:**
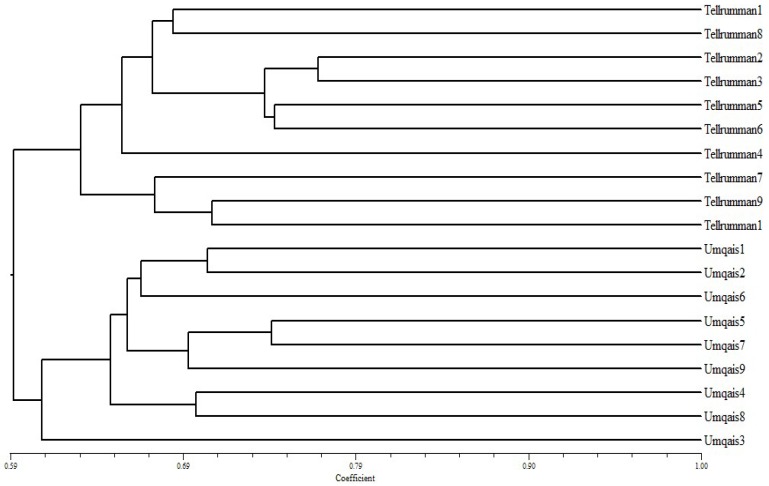
A dendrogram showing relationships among 19 Jordanian *Pleurotus* isolates by using ISSR markers data and the Dice genetic similarity and the Un-weighted pair group method with arithmetic average (UPGMA) clustering method.

The molecular data was used to build a dendrogram based on Dice’s coefficient of similarity to assess the genetic relatedness between the collected 19 *Pleurotus* isolates (Figure 4). The genetic similarity among the 19 isolates ranged from 59% to 74%, indicating the existence of high genetic diversity between them and a clear clustering based on collection site was observed. At the Tell ar-Rumman location, isolates #2 and #3 were closely related to each other, while, at Um Qais location, isolates #5 and #7 were also closely related to each other in the second clade. Percentage of polymorphism among 19 isolates of *Pleurotus*, obtained by ISSR markers was high enough to discriminate the isolates according to their collection site in Jordan.

## 4. Discussion

In this study, 19 collected Jordanian *Pleurotus* isolates from two different habitats were examined for their morphological characteristics and their identities were analyzed by using different molecular markers. Sequence comparison of the ITS and nLSU regions is widely used in taxonomy and molecular phylogeny of fungi because it is easy to amplify even from small quantities of DNA due to the high copy number of rDNA genes with a high degree of variation even between closely related species [22,29]. In this study, the amplification of ITS regions from different Jordanian isolates gave band sizes of around 600 bp; this is in general agreement with Imtiaj et al. [13], who found that the ITS amplified products from *Pleurotus* isolates collected from different geographical regions showed minor differences in size. On the other hand, differences in sizes of ITS amplified products are considered a useful tool to discriminate between *Pleurotus* spp. collected from different geographical regions. For instance, a maximum length of ITS amplified products was recorded in *P. djamor* (667 bp), whereas the minimum length was recorded in *P. ferulae* (571 bp) [30]. *P. nebrodensis, P. eryngii*, and *P. cornucopiae* produced identical sizes for ITS products (639 bp) but still the nucleotide sequences were different. The sequence analysis of ITS and nLSU regions in the 19 Jordanian *Pleurotus* isolates showed that all collected isolates produced high similarity percentages (above 97%) with ITS and nLSU sequences from *P. eryngii*. Phylogenetic analysis confirmed that the 19 Jordanian *Pleurotus* isolates grouped with *P. eryngii var. erygnii-ferulae* confirming their identity within the complex [27] (Figure 2). However, caution should be considered in interpreting rDNA sequence data analysis as closely related *Pleurotus* spp. could not be distinguished by ITS analysis alone and further sequence analysis using other nuclear genes such as *β-tubulin*, *RPB2*, and *EF1α* might be needed [31,32,33]. For instance, sequence variations in *EF1-α* gene were able to discriminate the *P. nebrodensis* from China and Sicily from *P. eryngii-ferulae* from the Mediterranean region [33].

In this study, the 19 Jordanian strains showed high levels of genetic diversity as revealed by using ISSR markers. The PIC percentages were considerably high, which is in agreement with previous findings [8,29]. For instance, the genetic diversity in 15 Chinese *P. pulmonarius* by using 20 ISSR primers generated 283 bands from which 211 were polymorphic (74.6%), which is in agreement with our findings [8]. In another study, Prasad and Agarwal [7] used SSR and ISSR markers for discrimination between different commercial mushroom samples (*A. bisporus*; *P. eryngii*; *L. edodes*; *H. tessellatus*; *P. ostreatus* and *P. diamor*), and ISSR markers were found to be powerful in discriminating between mushroom species. RAPD and ISSR were found to be more useful in distinguishing *P. eryngii* isolates and varieties for mushroom breeding programs [15,28]. For instance, RAPD analysis was used to assess the genetic diversity among 45 *Pleurotus* strains collected from five different host-plants in Iran, which resulted in clustering the isolates based on their host-plants [34]. The ISSR markers used in this study were used previously to assess genetic diversity among 32 *H. marmoreus* isolates from different locations in China [28]. The average number of amplified bands per primer was 11.0 and the polymorphism information content was 87.4%, indicating a high level of polymorphism among the tested markers, which is the case with our findings. The proportion of polymorphic bands ranged from 52.9% (primer UBC899) to 100% (primers UBC823, UBC824, UBC840, UBC841, UBC842, and UBC900), with an average of 87.46%. In another study, Ravash et al. [34], used ISSR and ScoT markers to evaluate the genetic diversity of the wild mushroom (*P. eryngii var. tuoliensis*) and they concluded that a high level of genetic polymorphism (96.3%) exists. Similar results were obtained with other edible species such as *Lentinula edodes* (99.6%) [35] and *Auricularia polytricha* (99.8%) [36]. In contrast, Yin et al. [17] found that RAPD markers were the best in differentiation of 15 *P. pulmonarius* isolates in China, with a polymorphic percentage of 79.5% as compared to 74.6% using ISSR and 74.8% by using SRAP marker.

In this study, NTSys analysis indicates an association of collected isolates to their geographical distribution and collection sites (Figure 4). Such results were observed in wild *P. eryngii var. tuoliensis* from china where a clear clustering of collected isolates was obtained [37]. The geographic distribution among the two habitats in Jordan is clearly obvious where a clear difference in altitude, climate, and rainfall exist. In addition, the degree of differentiation between populations may be affected by many factors such as gene flow, genetic drift, and geographical distribution [33]. However, clear genetic variations exist between collected isolates within each site. Furthermore, the samples that were distinctly less similar with *P. eryngii* in ITS and nLSU sequence analysis didn’t form any outgroup as expected and clustered with isolates from the same location. This might be explained that higher levels of gene flow might lead to less variation among local populations. For instance, longitudinal differences between different habitats played an important role in the degree of polymorphism among *P. eryngii* isolates in Israel [38].

## 5. Conclusions

In this study, the morphological characterization of 19 Jordanian *Pleurotus* isolates collected from two different sites indicate differences in cap color and diameter, gills color, shape of the stem, and spore measurements. Molecular identification of the Jordanian isolates using ITS and nLSU sequence analysis demonstrated that the majority of them share high sequence similarity with *P. eryngii.* ISSR markers used in this study generated high PIC values indicating the existence of a high degree of genetic diversity among *Pleurotus* isolates. Phylogenetic analysis revealed a clear association with geographical distribution and collection sites. In future, selection for suitable techniques for a large scale production and commercialization accompanied with breeding strategies with strains of other related populations is needed for king oyster mushroom in Jordan.

## Figures and Tables

**Table 1:** Morphological characteristics of mature *Pleurotus* Mushroom’s Fruit Bodies and spores collected naturally from two different locations across Jordan.

Item	Description	Um-Qais	Tell ar-Rumman
Cap	Color	White, gray to beige	Flesh white to grey brown
	Shape	Funnel shaped and irregular	Funnel shaped and irregular
	Diameter	3–9 cm	4–9 cm
Stem	Color	Flesh white	White to brown
	Shape	Eccentric thick, cylinder shape	Thick cylindrical
	Dimensions	Height:1–4 cm; Diameter: 1 cm	Height:1–4 cm; Diameter: 1 cm
Spore	Color	White, creamy to yellow	White, creamy to yellow
	Shape	Elliptic narrow; cylinder shape	Elliptic narrow, cylinder shape
	Print	White	White
	Measurements	Length: 5–7 µm; Width: 2–3 µm	Length: 5–10 µm; Width: 2–5 µm
Gills	Color	Greyish	Whitish honey
	Shape	Long extended internally to stem	Long extended internally to stem
	Attachment	Descending with stem	Descending with stem
Flesh	Color and smell	White with taste and smell pleasant	White with taste and smell pleasant

**Table 2:** Molecular identification of 19 Jordanian *Pleurotus* isolates using internal transcribed spacer (ITS) and nuclear large subunit (nLSU) rRNA sequence analysis.

Isolates		Best nr Database Similarity Hit Using nLSU			Best nr Database Similarity Hit Using ITS		
No	Code *	Taxon	ID%	GenBank Accession No.	Taxon	ID%	GenBank Accession No.
1	**Q1**	*P. eryngii*	98	KY963032.1	*P. eryngii var. ferulae*	99	KY962437.1
2	**Q2**	*P. eryngii*	99	AB777519.1	*P. eryngii*	97	KY962448.1
3	**Q3**	*P. eryngii*	99	AY450347.1	*P. eryngii*	99	KX977448.1.1
4	**Q4**	*P. eryngii*	100	AB777519.1	*P. eryngii*	99	MG282489.1
5	**Q5**	*P. eryngii*	99	AY450347.1	*P. eryngii var. ferulae*	99	MG282459.1
6	**Q6**	*P. eryngii*	99	AY450347.1	*P. eryngii*	97	KX977448.1
7	**Q7**	*P. eryngii*	99	AB777519.1	*P. eryngii*	99	MG282489.1
8	**Q8**	*P. eryngii*	99	KY963084.1	*P. eryngii*	99	KX977448.1
9	**Q9**	*P. eryngii*	99	KY963084.1	*P. eryngii*	98	JX429941.1
10	**R1**	*P. eryngii*	99	AB777519.1	*P. eryngii*	100	MG282489.1
11	**R2**	*P. eryngii*	99	AB777519.1	*P. eryngii*	99	MG282489.1
12	**R3**	*P. eryngii*	99	MG282549.1	*P. eryngii*	100	MG282489.1
13	**R4**	*P. eryngii*	99	MG282549.1	*P. eryngii*	99	KX836359.1
14	**R5**	*P. eryngii*	99	MG282549.1	*P. eryngii*	98	MG282489.1
15	**R6**	*P. eryngii*	99	MG282549.1	*P. eryngii*	99	MG282489.1
16	**R7**	*P. eryngii*	98	MG282549.1	*P. eryngii*	99	MG282489.1
17	**R8**	*P. eryngii*	98	MG282549.1	*P. eryngii*	99	MG282489.1
18	**R9**	*P. eryngii*	99	MG282549.1	*P. eryngii*	99	MG282489.1
19	**R10**	*P. eryngii*	99	MG282549.1	*P. eryngii*	99	MG282489.1

* Location codes: (Q) Um-Qais; (R) Tell ar-Rumman.

**Table 3:** ISSR markers analysis and polymorphism in 19 Jordanian *Pleurotus* isolates.

Primer	Sequences	Total Bands	Polymorphic Bands	PIC (%)
UBC807	(AG)8T	14	10	71.4
UBC808	(AG)8C	11	9	81.8
UBC809	(AG)8G	15	1	6.66
UBC810	(GA)8T	12	9	75
UBC811	(GA)8C	0	0	0
UBC812	(GA)8A	11	10	90.9
UBC816	(CA)8T	11	8	72.7
UBC823	(TC)8C	16	13	81.2
UBC825	(AC)8T	12	11	91.6
UBC826	(AC)8C	12	8	66.6
UBC827	(AC)8G	13	12	92.3
UBC864	(ATG)5	9	7	77.7
UBC866	(CTC)6	8	5	62.5
UBC868	(GAA)6	9	8	88.8
UBC873	(GACA)4	7	4	57.1
UBC874	(CCCT)4	9	8	88.8
UBC876	(GATA)2(GACA)2	12	11	91.66
UBC880	(GGAGA)3	15	11	73.3
Total		196	141	
Mean		10.89	7.83	70.56

## Abbreviations

BLAST	Basic Local Alignment Search Tool
ISSR	Inter simple short repeat
ITS	Inter transcribed spacer
PDA	Potato dextrose Agar
BlASTn	nucleotide-nucleotide blast
NTSys	Numerical Taxonomy Multivariate Analysis System
PIC	Polymorphism Information Content
UPGMA	Unweighted Pair Group Method with Average
